# Genome-wide expression differences in anti-*Vegf* and dexamethasone treatment of inflammatory angiogenesis in the rat cornea

**DOI:** 10.1038/s41598-017-07129-4

**Published:** 2017-08-15

**Authors:** Pierfrancesco Mirabelli, Anthony Mukwaya, Anton Lennikov, Maria Xeroudaki, Beatrice Peebo, Mira Schaupper, Neil Lagali

**Affiliations:** Department of Ophthalmology, Institute for Clinical and Experimental Medicine,Faculty of Health Sciences, Linkoping University, 58183 Linköping, Sweden

## Abstract

Angiogenesis as a pathological process in the eye can lead to blindness. In the cornea, suppression of angiogenesis by anti-VEGF treatment is only partially effective while steroids, although effective in treating inflammation and angiogenesis, have broad activity leading to undesirable side effects. In this study, genome-wide expression was investigated in a suture-induced corneal neovascularization model in rats, to investigate factors differentially targeted by dexamethasone and anti-*Vegf*. Topical treatment with either rat-specific anti-*Vegf*, dexamethasone, or normal goat IgG (sham) was given to sutured corneas for 48 hours, after which *in vivo* imaging, tissue processing for RNA microarray, and immunofluorescence were performed. Dexamethasone suppressed limbal vasodilation (P < 0.01) and genes in PI3K-Akt, focal adhesion, and chemokine signaling pathways more effectively than anti-*Vegf*. The most differentially expressed genes were confirmed by immunofluorescence, qRTPCR and Western blot. Strong suppression of *Reg3g* and the inflammatory chemokines *Ccl2* and *Cxcl5* and activation of classical complement pathway factors *C1r, C1s*, *C2*, and *C3* occurred with dexamethasone treatment, effects absent with anti-*Vegf* treatment. The genome-wide results obtained in this study provide numerous potential targets for specific blockade of inflammation and angiogenesis in the cornea not addressed by anti-*Vegf* treatment, as possible alternatives to broad-acting immunosuppressive therapy.

## Introduction

Angiogenesis is an essential physiologic process occurring during embryogenesis as well as in adult organisms in the context of wound healing, muscle growth, and the menstrual cycle. Pathologic angiogenesis, however, is involved in multiple disorders including ocular, inflammatory, and cardiovascular diseases as well as in cancer^1, 2^. In the eye, pathologic invasion of blood vessels into the normally avascular cornea secondary to inflammation can cause blindness due to fibrosis and edema disrupting corneal transparency^3^. Similarly, choroidal and retinal neovascularization can lead to blindness in wet age-related macular degeneration (AMD)^4^, proliferative diabetic retinopathy^5^, retinopathy of prematurity^6^ and uveitis^7^. Similar to hypoxia, inflammation is considered a major trigger of angiogenesis and its biochemical cascades are closely linked with angiogenic pathways^8^. Vascular endothelial growth factor A (VEGFA), which disrupts blood vessel walls and stimulates the growth of new vessels^9^, is a central mediator in both inflammation and angiogenesis. VEGFA is secreted by corneal epithelium^10^, infiltrating leukocytes (myelomonocytes and neutrophils)^11–13^, and vascular endothelial cells^14^ and its expression is upregulated in the early phases of neovascularization^15^. Blockade of VEGFA has therefore been widely used therapeutically. Chimeric monoclonal antibodies against VEGFA (hereafter referred to as anti-VEGF) are an established treatment modality for different retinal diseases such as wet AMD^4^, although repeated treatments (typically monthly intravitreal injections) are required to sustain angiogenic suppression, and the therapy fails in at least 20% of cases^16, 17^. In oncology, anti-VEGF is used to augment treatment of many types of tumors, but its value is limited by resistance to anti-VEGF treatment that often develops over time^18–20^. Recently, anti-VEGF treatment has been introduced as an alternative for corneal neovascularization^21^. Only a partial reduction in corneal neovessels, however, has been achieved with anti-VEGF treatment given by topical^22^, subconjunctival^23^, intrastromal^24^, or intraocular^25^ routes, with a reduction in area of neovascularization in the range of 15–20% in experimental studies^26^, and 36–61% in clinical studies^22, 23, 27^.

Alternatively, topical steroids have historically been used widely in clinical practice to treat inflammation and corneal neovascularization^28, 29^. Steroids are also effective in the treatment of other ocular conditions related to high VEGF production, such as macular edema secondary to diabetic retinopathy^30^ or central retinal vein occlusion^31^. The efficacy of steroids, however, must be weighed against their many possible side effects, as local steroid use in the eye has been reported to cause secondary glaucoma, corneal thinning and perforation, cataract, and exacerbation or reactivation of herpes infection^28^.

Due to the risks associated with the use of immunosuppressive steroids on the one hand, and the limited efficacy of anti-VEGF treatment in suppressing angiogenesis on the other, alternative treatments are needed. Specifically, addressing the inflammatory component of the angiogenic response (as steroids do) but in a more targeted manner than steroids, may avoid side effects while potentially improving the efficacy of alternative treatments. With this rationale in mind, we previously reported on a comparison of topical anti-*Vegf* treatment with steroids (topical dexamethasone) in a model of inflammatory neovascularization in the rat cornea^32^. Dexamethasone dramatically suppressed angiogenesis, but a closer investigation of gene expression of selected inflammatory and angiogenic cytokines failed to explain the differential effect of the treatments^32^.

The purpose of the present study was to investigate the active phase of inflammation preceding sprouting angiogenesis in the rat cornea, when both inflammatory and angiogenesis-related factor expression are naturally strong^33^. During this early phase of inflammation, we performed genome-wide microarray analysis in anti-*Vegf* and dexamethasone-treated groups to systematically investigate the differential effect of these treatments at the gene expression level. The goal was to reveal candidate factors that could be targeted in the future to achieve a more effective suppression of inflammation and angiogenesis than is possible with anti-VEGF treatment, but in a more targeted manner than steroid therapy.

## Results

### Dexamethasone differentially suppresses early vessel dilation relative to anti-*Vegf* treatment but not inflammatory cell invasion or *Vegfa* expression in the cornea

**Figure 1:**
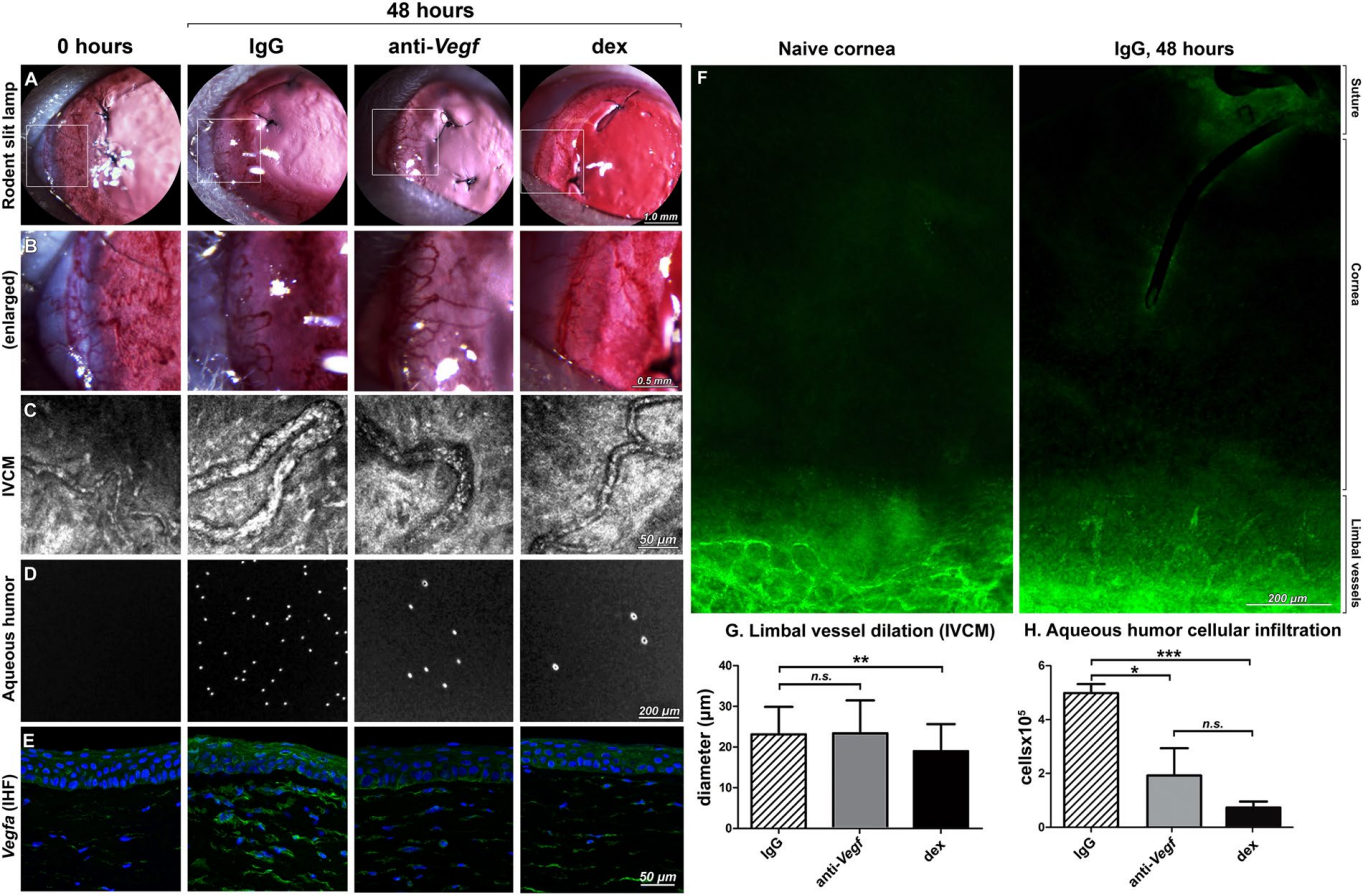
Limbal vessel dilation, inflammatory cell infiltration and *Vegfa* expression following IgG, anti-*Vegf* and dexamethasone treatment of suture-induced injury in the rat cornea. (**A**) Slit lamp photographs depicting limbal vessel dilation and looping angiogenesis^35^ 48 h after suture placement in IgG and anti-*Vegf* groups, not detected in the dexamethasone group; expanded view of the white boxed area in (**B**). (**C**) IVCM images of limbal vessel dilation, and the corresponding measurements of limbal vessel diameter in (**G**); (**D**) Microscopic images of aqueous humor cellular content, and the corresponding quantification in (**H**). (**E**) Immunofluorescence of *Vegfa* (green) and DAPI counterstaining of nuclei (blue) in corneal tissue sections. (**F**) CD31 staining (green) of naïve and sutured cornea at 48 hours: the latter shows no sign of corneal neovascularization. n = 4 corneas per group for (**G)** and (**H)**, *P < 0.05; **P < 0.01; ***P < 0.001; n.s. = P > 0.05 and error bars represent SD.

At 48 h after suture placement in the temporal rat cornea, no new vessel sprouts were observed (Fig. 1A,B,F); this result was expected at this early time point. According to our prior work, sprouting is initiated at day 3–4 in our model^34^. Dilation of limbal vessels, however, was noticeable in IgG and anti-*Vegf* treatment groups at 48 h, while dexamethasone effectively suppressed vasodilation. This result was found by direct measurement of limbal vessel diameter from *in vivo* confocal microscopy (IVCM) images. The decrease in vasodilation was significant (P < 0.01) upon dexamethasone treatment (Fig. 1C,G).

Inflammatory cell infiltration into the cornea, however, was not suppressed by dexamethasone treatment. CD45-positive inflammatory leukocytes invaded all sutured corneas regardless of treatment (Supplementary Figure 1). Infiltrating cells were also observed by IVCM in all sutured corneas, and quantitative analysis demonstrated no significant difference in inflammatory cell density at 48 h in any of the treatment groups (Supplementary Figure 1).

A prominent inflammatory response was also observed in the aqueous humor. While aqueous humor from non-sutured rat eyes is acellular, many cells infiltrated the aqueous humor in the IgG group. Anti-*Vegf* and dexamethasone both significantly reduced (P < 0.001) the number of cells in the aqueous humor (Fig. 1D,H), indicating an effect of topical treatment deeper into the eye, but similar to the corneal stroma there was no significant difference in inflammatory cell recruitment between anti-*Vegf* and dexamethasone groups.

The expression of *Vegfa* ligand in the corneal stroma was partially suppressed by both anti-*Vegf* and dexamethasone relative to IgG (Fig. 1E), with further epithelial expression effectively suppressed by anti-*Vegf*. Total protein expression in the corneal tissue examined by Western blot confirmed a partial suppression of *Vegfa* (Supplementary Figure 2), thus confirming the expected effect of the topical antibody.

### Differentially expressed genes (DEGs) in key biological processes and pathways are preferentially suppressed in dexamethasone versus anti-*Vegf* treatment

**Figure 2:**
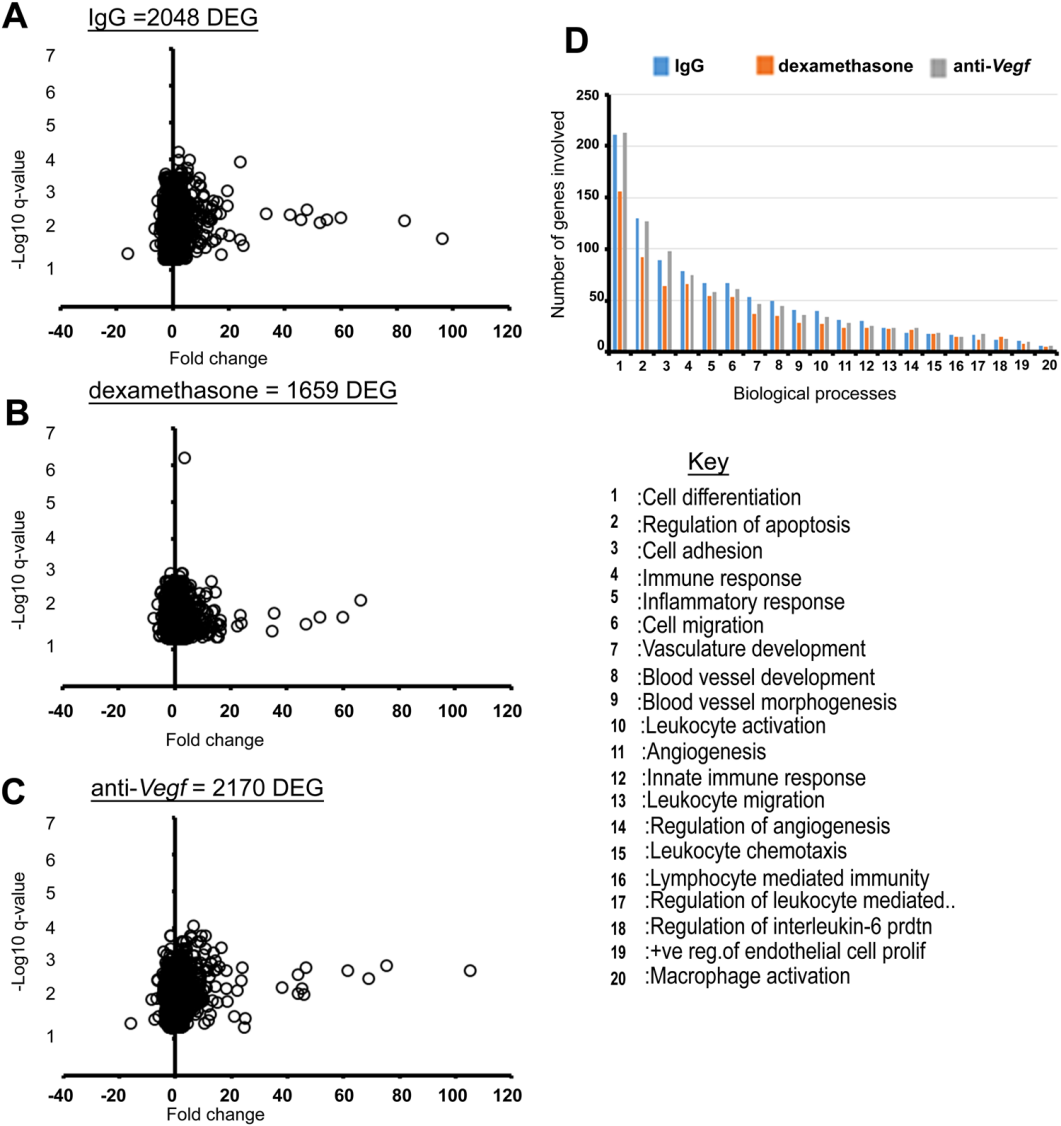
Differentially expressed genes (DEGs) in IgG, dexamethasone and anti-*Vegf* treated groups, and selected enriched biological processes. (**A–C)** Volcano plots of the DEGs in IgG, dexamethasone and anti-*Vegf* groups respectively. **(D)** Selected biological processes of interest, indicating the number of DEGs involved in the processes for each treatment.

Despite effectively suppressing *Vegfa* expression in the corneal tissue, anti-*Vegf* treatment is unable to suppress corneal angiogenesis as efficiently as dexamethasone after 7 days of treatment, as previously shown^32^. To investigate factors suppressed by dexamethasone but not by anti-*Vegf* treatment, a genome-wide expression analysis using microarrays was performed 48 h after suture placement in IgG, dexamethasone, and anti-*Vegf* treated groups. In the naïve group, no sutures were placed into the cornea. After filtering the gene expression data, a total of 2048, 1659 and 2170 genes were differentially expressed in IgG, dexamethasone, and anti-*Vegf* treated groups, respectively, relative to naïve (control) corneas (Fig. 2A–C). Next, the DEGs were classified into biological processes, and representative biological processes were compared across treatments with respect to the number of DEGs involved in each process (Fig. 2D). In almost all key biological processes regulating the inflammatory response and angiogenesis, dexamethasone had fewer DEGs represented than in IgG or anti-*Vegf* groups. Only marginal differences were noted between IgG and anti-*Vegf*.

Following biological process enrichment analysis, the top five biological processes from Fig. 2D (i.e., cell differentiation, regulation of apoptosis, cell adhesion, immune response, and inflammatory response) were analyzed, and the DEGs involved in these processes were identified. The difference in fold change expression (relative to the naïve cornea) between dexamethasone and anti-*Vegf* was then determined. The top 10 up- and downregulated genes in terms of fold change difference (P < 0.05 between dexamethasone and anti-*Vegf*) are presented in Table 1a.

**Table 1:** The top 10 differentially up- and downregulated genes involved in selected biological processes (a), and in selected pathways (b).

a: From biological process enrichment analysis					b: From pathway enrichment analysis				
Gene ID	FC:Dex	FC: anti-*Vegf*	FC:Diff	P-Value	Gene ID	FC:Dex	FC:anti-*Vegf*	FC:Diff	P-Value
Top 10 genes differentially upregulated by dexamethasone					Top 10 genes differentially upregulated by dexamethasone				
*C3*	22.12	5.56	16.56	1.04E-02	*C3*	22.12	5.56	16.56	1.04E-02
*Ctgf*	1.31	−2.95	4.26	2.04E-03	*C1s*	8.5	4.39	4.11	2.25E-03
*C1s*	8.5	4.39	4.11	2.25E-03	*Serping1*	8.34	5	3.34	1.41E-02
*Serping1*	8.34	5	3.34	1.41E-02	*Tgfb2*	1.06	−1.92	2.99	3.27E-03
*Tgfb2*	1.06	−1.92	2.99	3.27E-03	*Fgfr1*	1.64	−1.12	2.76	2.60E-05
*Pou3f3*	1.13	−1.61	2.74	1.12E-03	*Mpzl1*	1.09	−1.59	2.69	1.77E-03
*Tfrc*	*3.64*	1.8	1.84	2.76E-03	*Pdgfa*	1.56	−1.01	2.58	1.08E-04
*Enpp2*	−1.17	−2.72	1.55	1.61E-02	*Mmp9*	4.02	1.92	2.09	8.22E-03
*Cd36*	3.35	1.82	1.53	7.12E-03	*C1r*	4.88	3.16	1.71	6.38E-03
*Cdh2*	−1.39	−2.79	1.4	4.78E-03	*Col6a1*	−1.2	−2.81	1.62	1.28E-03
**Top 10 genes differentially downregulated by dexamethasone**					**Top 10 genes differentially downregulated by dexamethasone**				
*Ccl2*	66.17	104.94	−38.76	1.37E-02	*Ccl2*	66.17	104.94	−38.76	1.37E-02
*Reg3g*	2.65	18.18	−15.53	3.35E-03	*Cxcl5*	6.89	14.53	−7.64	3.33E-02
*Cxcl5*	6.89	14.53	−7.64	3.33E-02	*Cfi*	3.95	8.64	−4.69	1.25E-02
*Cfi*	3.95	8.64	−4.69	1.25E-02	*Pik3r3*	−1.52	1.2	−2.73	1.02E-05
*Klk6*	−1.04	2.1	−3.14	1.49E-02	*Prkx*	−1.02	1.56	−2.58	3.38E-03
*Adam10*	−1.02	1.96	−2.98	3.18E-03	*Cntf*	1.3	2.46	−1.16	8.44E-03
*Cast*	−1.2	1.78	−2.98	2.77E-04	*Cldn1*	1.38	2.51	−1.13	3.98E-03
*Klk8*	1.06	3.96	−2.9	5.25E-03	*Nox1*	−3.88	−2.82	−1.07	3.47E-02
*Trim54*	−1.08	1.71	−2.79	2.28E-03	*C1qa*	2.55	3.62	−1.07	4.16E-02
*Ehf*	−1.11	1.68	−2.79	4.66E-04	*Ccnd2*	1.94	2.83	−0.89	1.23E-02

The abbreviation’FC Diff’ represents fold change in dexamethasone minus fold change in the anti-*Vegf* group. P-values are for the comparison dexamethasone versus anti-*Vegf*.

**Figure 3:**
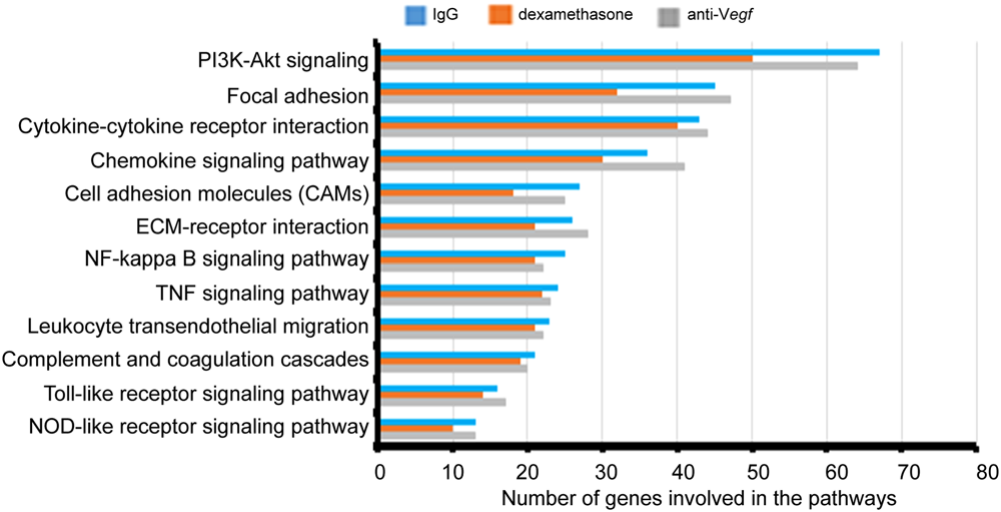
Pathway enrichment analysis indicates that dexamethasone suppresses most DEGs in all selected inflammatory and angiogenesis pathways, relative to IgG and anti-*Vegf* treatment.

DEGs were also classified into pathways. Pathway enrichment analysis indicated a reduction in the number of genes in all selected pathways in the dexamethasone group compared to IgG and anti-*Vegf* groups (Fig. 3). Genes in pathways such as PI3K-Akt signalling, focal adhesion, and chemokine signalling were suppressed the most by dexamethasone relative to anti-*Vegf*. IgG and anti-*Vegf* groups, however, had similar numbers of DEGs in the enriched pathways.

Among the DEGs involved in the selected pathways, the genes that were differentially expressed between dexamethasone and anti-*Vegf* groups (P < 0.05) are summarized in Table 1b. The number of genes active in the various pathways and their false discovery rate are given in Supplementary Table 1, and an extended version of the gene lists in Table 1 is given in Supplementary Table 2.

### Dexamethasone suppresses chemokines and enhances specific complement factors relative to anti-*Vegf* treatment

**Figure 4:**
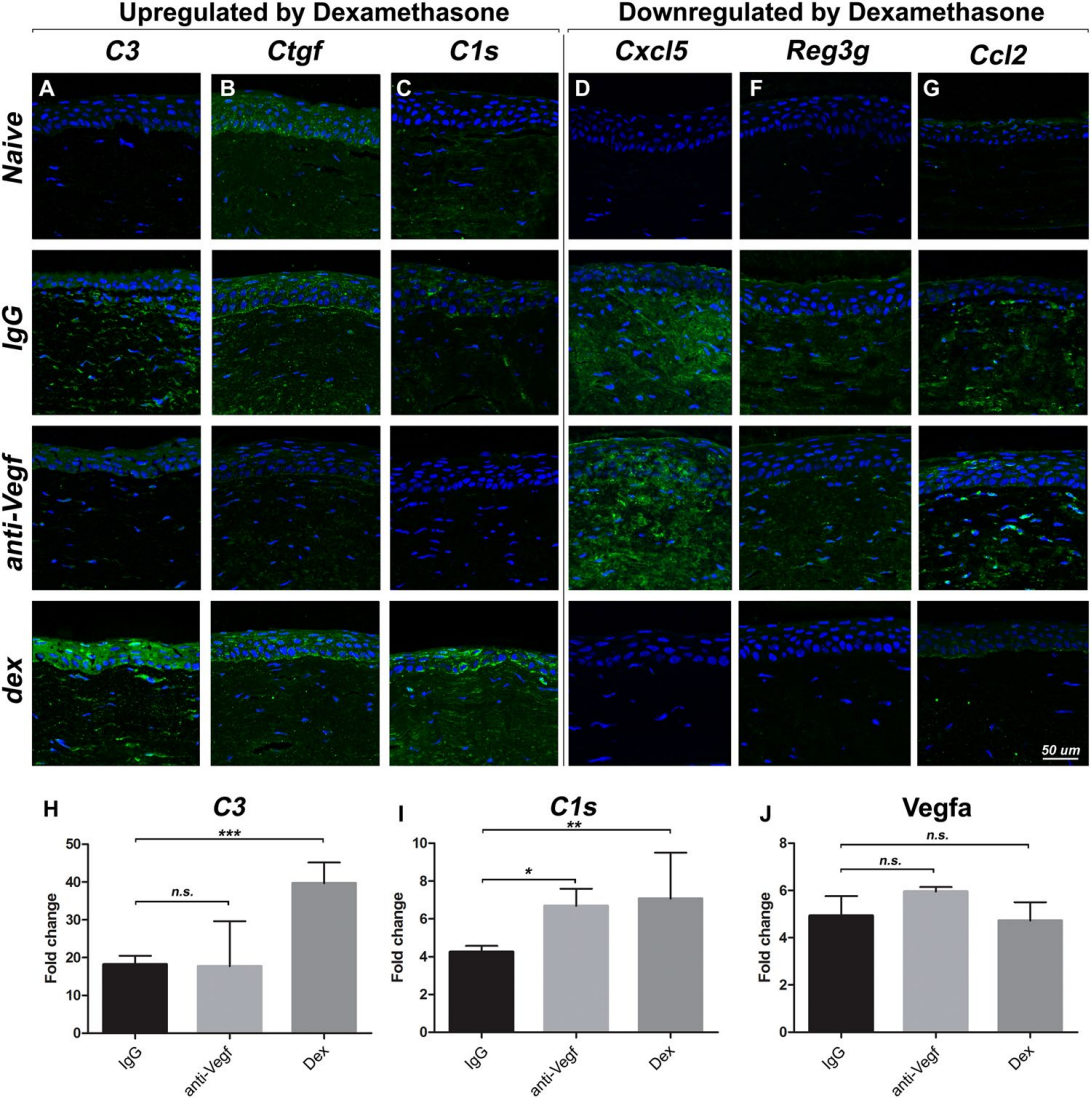
(**A–G**): Immunofluorescent staining of naïve, and sutured corneas from treatment groups (48 h post-suture). Factors most differentially regulated between dexamethasone and anti-*Vegf* groups from microarray analysis are shown. Expression signals are indicated in green, and DAPI counterstained nuclei in blue. Scale bar = 50 µm. (**H–J**): qPCR analysis of *C3*, *C1s* and *Vegfa* gene expression. The error bars represent SEM.

Among the genes presented in Table 1, *C3*, *Ctgf*, *C1s*, *Cxcl5*, *Reg3g*, and *Ccl2* were the most differentially regulated genes, with *C3*, *Ctgf* and *C1s* upregulated by dexamethasone, and *Cxcl5*, *Reg3g* and *Ccl2* downregulated by dexamethasone, both relative to anti-*Vegf*. Localization of their protein expression in the corneal tissue was evaluated by immunofluorescence (Fig. 4A–G), and was further supported by qPCR analysis (Fig. 4H–J). Pro-inflammatory factors *Cxcl5*, *Reg3g*, and *Ccl2*, not expressed in the naïve cornea, were expressed in the corneal tissue post-injury (IgG group). Anti-*Vegf* treatment did not affect this expression, whereas dexamethasone dramatically suppressed the expression of these factors in the tissue. Complement factor *C3*, similar to the pro-inflammatory factors, was not expressed in naïve corneas and moderately expressed in the IgG group post-injury. In contrast to the pro-inflammatory factors, however, anti-*Vegf* suppressed *C3* expression while dexamethasone enhanced it. Similarly, *C1s* was moderately expressed in naïve and injured corneas, with anti-*Vegf* treatment suppressing *C1s* and dexamethasone enhancing its expression in the tissue. *Ctgf*, or connective tissue growth factor, whose gene expression was also significantly enhanced by dexamethasone relative to anti-*Vegf*, showed enhanced protein expression in the epithelium and stroma with dexamethasone treatment relative to anti-*Vegf*.

As complement factor activation was observed with dexamethasone – a treatment normally known to suppress inflammation, complement proteins *C3* and *C1s* were further evaluated for protein expression in the tissue by Western blot analysis. Western confirmed the suppression of these factors by anti-*Vegf* treatment and enhanced expression with dexamethasone treatment (Supplementary Figure 2).

Finally, gene expression in the dexamethasone group was compared to the gene expression in the IgG (positive control) group. This was achieved by computing the fold change difference in expression between these groups (i.e. Fold change Dexamethasone minus Fold change IgG) (Supplementary Table 3). Relative to IgG treatment, dexamethasone highly suppressed pro-inflammatory factors including *Cxcl5* and *Reg3g*, but also *Serpinb2*, one of the top 5 factors downregulated during the endogenous restoration of corneal avascularity^33^. Conversely, complement factors *C2* and *C3* were among the most highly upregulated genes, also indicating an activation of complement by dexamethasone relative to the IgG treated group (Supplementary Table 3).

### Dexamethasone specifically activates the classical complement pathway promoting leukocyte recruitment, while anti-*Vegf* treatment does not

**Figure 5:**
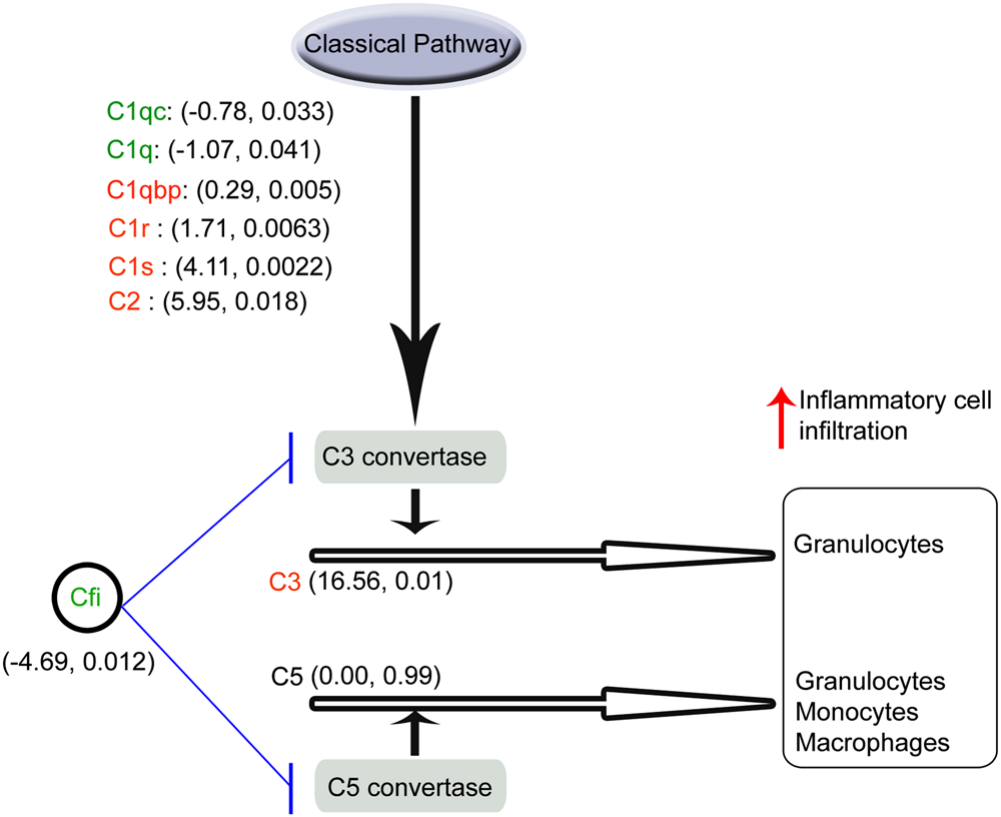
Summary of complement genes differentially regulated by dexamethasone treatment relative to anti-*Vegf*, and their roles in the complement cascade based on gene expression microarray analysis. Differentially regulated genes (upregulated genes labelled in red, downregulated genes in green) are shown along with their fold change difference (dexamethasone minus anti-*Vegf*) and corresponding p-value (between dexamethasone and anti-*Vegf* fold change) given in parentheses. All differentially regulated genes mapped to the classical pathway, while *Cfi*, a natural inhibitor of *C3* and *C5* convertase production, was strongly downregulated. This, combined with strong upregulation of *C3*, promoted pathways leading to inflammatory cell activation and invasion into the cornea following dexamethasone treatment. Further downstream complement components (*C6*–*C9*) leading to cell lysis were not differentially regulated by dexamethasone. The figure is an adapted version of the conceptual diagram presented by del Zoppo^37^.

Among the most differentially regulated genes, *Cfi* was one of the most suppressed by dexamethasone (Table 1), while *C1s*, *C2*, and *C3* were enhanced relative to IgG treatment (Supplementary Table 3). Because of the involvement of multiple complement genes, microarray data for genes of the complement cascade were examined in detail (Table 2). Nine complement genes were differentially regulated by dexamethasone, relative to anti-*Vegf*, all of these being confined to the early complement cascade (*C1 – C3*), in addition to *Cfi*. Among these, *C2* was one of the most strongly upregulated genes, not originally detected in the biological process and pathway analysis in Table 1. Dexamethasone, however, did not have a differential effect in expression of factors downstream in the complement cascade. The differentially regulated genes instead all mapped to the classical pathway of complement (Fig. 5), or as in the case of *C3* are common to multiple cascades. Furthermore, *Cfi* (an inhibitor of *C3* and *C5* convertases) was downregulated thereby promoting *C3* and *C5* pathways leading to inflammatory leukocyte invasion^36^ of the tissue.

**Table 2:** Summary of genes involved in the complement cascade from gene microarray analysis, and the FC Diff and the corresponding P values.

Gene ID	Gene Title	FC Diff: dex- anti-*Vegf*	P-Value dex vs anti-*Vegf*
***C1qa***	**complement component 1, q subcomponent, A chain**	**−1.07**	**4.16E-02**
***C1qc***	**complement component 1, q subcomponent, C chain**	**−0.78**	**2.92E-02**
***C1qbp***	**complement component 1, q subcomponent binding protein**	**0.29**	**4.12E-02**
***C1qtnf7***	**C1q and tumor necrosis factor related protein 7**	**0.39**	**4.85E-02**
***C1s***	**complement component 1, s subcomponent**	**4.11**	**2.25E-03**
***C1r***	**complement component 1, r subcomponent**	**1.71**	**6.38E-03**
***C2***	**complement component 2**	**5.95**	**1.87E-02**
***C3***	**complement component 3**	**16.56**	**1.04E-02**
***Cfi***	**complement factor I**	**−4.69**	**1.25E-02**
*C1ql1*	complement component 1, q subcomponent-like 1	−0.12	1.87E-01
*C1qb*	complement component 1, q subcomponent, B chain	−0.48	2.14E-01
*C1ql3*	complement component 1, q subcomponent-like 3	0.18	7.29E-01
*C1qtnf1*	C1q and tumor necrosis factor related protein 1	2.19	7.03E-02
*C1qtnf2*	C1q and tumor necrosis factor related protein 2	0.15	1.63E-01
*C1qtnf4*	C1q and tumor necrosis factor related protein 4	−2.12	8.54E-02
*C1qtnf5*	C1q and tumor necrosis factor related protein 5	0.26	7.65E-02
*C1qtnf6*	C1q and tumor necrosis factor related protein 6	−2.04	6.18E-01
*C3ar1*	complement component 3a receptor 1	−0.25	6.83E-02
*C4a*	complement component 4 A	0.10	1.68E-01
*C4bpa*	complement component 4 binding protein, alpha	−0.18	5.64E-01
*C4bpb*	complement component 4 binding protein, beta	−2.05	4.17E-01
*C5*	complement component 5	0.00	9.90E-01
*C5ar1*	complement component 5a receptor 1	0.80	1.14E-01
*C6*	complement component 6	−0.94	1.30E-01
*C7*	complement component 7	−2.09	3.25E-01
*C8a*	complement component 8, alpha polypeptide	2.01	8.76E-01
*C8b*	complement component 8, beta polypeptide	0.01	8.51E-01
*C8g*	complement component 8, gamma polypeptide	−0.03	7.41E-01
*C9*	complement component 9	0.00	9.83E-01
*Cfh*	complement factor H	0.50	1.87E-01
*Cfh/LOC100361907*	complement factor H/complement factor H-related protein B	2.14	1.09E-01
*Cfhr1*	complement factor H-related 1	0.10	5.45E-02
*Cfp*	complement factor properdin	2.12	2.08E-01
*Cr1l*	complement component (3b/4b) receptor 1-like	−0.03	5.05E-01
*Cr2*	complement component (3d/Epstein Barr virus) receptor 2	0.02	6.33E-01

Highlighted genes are those significantly different between treatments.

The expression profile for *C3*, *C1s* and *Vegfa* in all the treatment groups (IgG, anti-*Vegf* and dexamethasone) was verified by qPCR, confirming the trend of gene expression as shown by microarray, immunohistochemistry and Western blot analysis.

## Discussion

The cornea, a normally avascular tissue, maintains a tightly regulated and delicate balance between proangiogenic and antiangiogenic factors for it to stay avascular and transparent^38^. This balance can be disrupted, however, by pathologies such as injury, infection, or activation of an immune response, where inflammation plays a key role in modulating the angiogenic balance. Global gene expression analysis was performed in this study in a model of inflammation leading to angiogenesis, i.e., dilation and looping/pulling of pre-existing limbal vessels at 48 h post-injury, that in a later phase^15, 39^ is followed by angiogenic sprouting. In this model, just 48 h after injury, the number of genes differentially expressed relative to a naïve cornea was highest in the group treated with topical rat anti-*Vegf* antibody. Anti-*Vegf* treatment perturbed 122 more genes than injured control corneas and 511 more genes than dexamethasone treated corneas. This trend persisted at the biological process and pathway levels, with dexamethasone consistently suppressing more genes related to inflammation and angiogenesis processes and pathways than anti-*Vegf* treatment. The broad immunosuppressive activity of dexamethasone is known; however, much less is known about the specific factors beneficially influenced by dexamethasone in the context of corneal inflammation. Further analysis of processes and pathways revealed a number of genes with a high differential expression between dexamethasone and anti-*Vegf*, and a subset of these could be responsible for the potency of dexamethasone in suppressing corneal neovascularization. Six genes highly differentially expressed between these groups were *Cxcl5, Ccl2, Reg3g, C3, C1s*, and *Ctgf*. The protein product of these genes mirrored the gene expression characteristics in corneal tissue sections, as well as in Western blot of corneal tissue for *C3* and *C1s*, supporting the trend of the microarray gene expression analysis data. Moreover, these differences were present despite effective suppression of *Vegfa* by the anti-*Vegf* treatment at the protein level.

Interestingly, *Cxcl5, Reg3g*, and *Ccl2* were also three of the four most significantly suppressed genes during the endogenous restoration of corneal avascularity as we reported in a recent study^33^. Dexamethasone in this sense may mimic or activate natural mechanisms for maintaining corneal avascularity, whereas anti-*Vegf* treatment is clearly deficient in this regard.

*Reg3g* (regenerating islet-derived protein 3 gamma) is known as a bactericidal peptide^40^, that has also recently been reported to exacerbate pancreatic cancer in a model of inflammation-associated cancer progression, by activation of STAT3 and Nuclear Factor kB (NFκB) signaling pathways^41^. Little is known about the effect of *Reg3g* on angiogenesis, but its dramatic suppression with dexamethasone treatment and in the endogenous restoration of corneal avascularity^33^ warrants further investigation. *Serpinb2* known as plasminogen activator inhibitor type 2 (or PAI-2) was also highly suppressed in the dexamethasone treated group compared to IgG; *Serpinb2* appears as one of the top 5 factors downregulated during the endogenous restoration of corneal avascularity^33^. It is known to be overexpressed in inflammation, where it is expressed by monocytes, macrophages and vascular endothelial cells^42^, although its role in the context of angiogenesis is less clear^43^.

CXCL5 (also known as ENA-78, epithelial-derived neutrophil-activating peptide 78) plays an important role in the recruitment and trafficking of neutrophils/granulocytes^44^ while CCL2 (also known as MCP-1, monocyte chemoattractant protein-1) is involved in monocyte recruitment^45, 46^. Apart from these pro-inflammatory functions, CXCL5 and CCL2 also have direct and potent effects on the migration and proliferation of vascular endothelial cells^47–50^. From our pathway analysis, dexamethasone differentially suppressed genes in both NFκB and Tumor Necrosis Factor (TNF) pathways. TNF is a key inflammatory mediator involved in NFκB activation, which in turn regulates the transcription of CXCL5 and CCL2^51^. Although dexamethasone effectively suppressed both *Cxcl5* and *Ccl2* in our model, *in vivo* imaging indicated the absence of an inhibitory effect on the early recruitment of inflammatory cells into the cornea, which was further confirmed by the observation of CD45 positive leukocytes in all treatment groups. This is in apparent contradiction to the inflammatory cell recruitment roles of CXCL5 and CCL2; however, complement activation provides a possible explanation for the observed inflammatory cell recruitment despite the effective suppression of the chemotactic stimulus by dexamethasone.

Both microarray gene expression data and immunofluorescence in corneal tissue sections indicated increased transcription and translation respectively, of complement factors *C3* and *C1s* in dexamethasone-treated corneas compared to anti-*Vegf*. Western blot analysis further confirmed this upregulation of complement factors. Increased gene expression of *C3* was reported after treatment of rat mesenchymal cells with dexamethasone or other glucocorticoids^52^, while in another study C3 enhancement occurred in vascular endothelial cells exposed to IL1α together with glucocorticoids, with glucocorticoids enhancing the IL1-α induced stimulation of C3 production^53^. In this study, deeper analysis of the microarray data for the complement cascade revealed a selective enhancement of a number of factors within the classical complement pathway including C3, and suppression of complement factor I expression by dexamethasone relative to anti-*Vegf*. Together, these events lead to an increased infiltration and activation of inflammatory cells^36^ (such as monocytes and neutrophils), in a manner apparently independent of CXCL5 and CCL2. Because of the additional potent pro-angiogenic properties of these chemokines, it could be hypothesized that dexamethasone suppressing CXCL5 and CCL2 achieves a potent anti-angiogenic effect, while instead activating the classical complement pathway in a chemokine-independent manner recruits inflammatory cells functional to mount an anti-microbial response. Notably, dexamethasone has been shown to promote antimicrobial activity in several strains of microorganisms *in vitro* ^54^. In the cornea, the complement system seems to be crucial for the protection against gram-negative bacteria such as Pseudomonas aeruginosa, as depletion of *C3* renders the normally resistant mouse strain DBA/2 susceptible to such infection^55^. Corneal fibroblasts in healthy human eyes have been shown to synthesize complement components, among them *C3*, and the complement cascade is believed to be activated chronically at a low level in the healthy cornea^56^. Verhagen *et al*. showed, however, that complement depletion in the cornea had almost no effect on the development of experimental keratitis and LPS-induced corneal inflammation^57^.

In the present model of corneal neovascularization, complement activation appears to be beneficial; in other contexts, however, complement might have several diverse and possibly opposite roles in different pathologic conditions, with a possible tissue- and context-specificity. The complement system is clearly involved in diseases characterized by pathologic angiogenesis; in age-related macular degeneration^58^ and laser-induced choroidal neovascularization in mice^59^, complement activation has been associated with increased disease severity. Much evidence exists that activation of complement is part of the pathogenesis of AMD, both in its dry and wet forms, and polymorphism of gene loci such as complement factor H and complement factor B greatly increases the risk of developing these diseases^60, 61^. In other pathologies, complement appears to have a more complicated and fine-tuned role: for example in cancer, it may have a role in promoting tumor angiogenesis on the one hand, while it exerts a cytotoxic effect on tumor cells on the other^62^. Langer *et al*. showed that in a murine model of oxygen-induced retinopathy, a model for retinopathy of prematurity, *C3* and *C5* deficient mice had a higher rate of pathologic vessel growth^63^. In that study, it was suggested that activation of the complement system prevents retinal neovascularization, a process that appears to be mediated by the polarization of macrophages towards an anti-angiogenic phenotype. Using similar models, recent studies found that the blockage of specific complement factors from the alternative pathway (such as *Cfb*) resulted in enhanced pathologic retinal neovascularization^64, 65^. These results, albeit in retinal models of hypoxia, are in line with our findings of higher expression of *C3* in corneas where the neovascular response is suppressed.

The specific role of the complement in corneal neovascularization, however, is still poorly understood due to the complexity of the system, its possible tissue-specific roles and the lack of reliable study models^66^. Further studies are required to clarify in detail the role of the various complement factors and other identified factors in angiogenesis, and to determine if selective inhibition or modulation of these factors could improve the treatment of inflammation and neovascularization.

## Conclusion

For the first time to our knowledge, a systematic investigation of genome-wide expression changes with different treatments for corneal inflammation and neovascularization was undertaken. Robust genome-wide analysis yielded candidate factors confirmed at the tissue and protein expression levels, providing gene lists as a resource for investigating novel targets for anti-inflammatory and anti-angiogenic therapy specific to the corneal milieu. Most notably, the chemokines CXCL5 and CCL2 are possible targets for inhibiting the early inflammatory response leading to corneal neovascularization – factors which anti-VEGF treatment does not influence. REG3G and SERPINB2 are similarly novel candidate targets in this respect. Additionally, activation of the complement pathway by dexamethasone, notably the classical cascade, was unexpected and the consequences of this activation in our model warrant further investigation. Finally, targeting inflammation-specific factors or complement in addition to VEGF may provide a route for improving the efficacy of anti-VEGF treatment while avoiding negative side effects of steroid use.

## Materials and Methods

### Research model

A previously described model of inflammatory neovascularization in the rat cornea was used with minor modifications^39^. In brief, two intra-stromal sutures were placed 1.5 mm from the temporal limbus of the right cornea to induce a robust inflammatory response a few hours after suturing^15, 34^. Here, animals were examined 48 hours after suturing, during the pre-sprouting phase.

### Animal care

Between twelve- to sixteen-week-old male Wistar rats weighing 300 to 400 g (Scanbur AB, Sollentuna, Sweden) were used. All animal experiments were conducted in accordance with the Guidelines for the Use of Animals in Ophthalmic and Vision Research of the Association for Research in Vision and Ophthalmology (ARVO), and with the guidelines set out in the EU Directive 2010/63/EU on the protection of animals used for scientific purposes. All procedures performed followed a protocol approved by the Regional Animal Ethics Review Board of Linköping, Sweden (application no. 7–13). Animals were maintained in a licensed care facility in standard conditions (Ventilation: 15 air changes per hour; temperature: 22 ± 2 °C; humidity: 55 ± 10%; 12 h light/dark cycle; background sound: less than 55 dB; cage volume: 1760 cm^2^ for two animals, with food and water given *ad libitum*).

Anesthesia and analgesia were performed by intraperitoneal injection of Ketanest (esketamine 25 mg/ml, 0.4 ml, Pfizer) and Dexdomitor (dexmedetomidine hydrochloride 0.5 mg/ml, 0.2 ml, Orion Pharma). Additionally, one drop of topical anesthesia (tetracaine hydrochloride 1%, Chauvin Pharmaceuticals, UK), was used. A subcutaneous injection of Antisedan (atipamezole 5 mg/ml, 0.1 ml, Orion Pharma) was used to reverse anesthesia. The same anesthetic procedure was used for *in vivo* examinations. At the experimental endpoint, euthanasia was achieved by intracardial injection of sodium pentobarbital 60 mg/ml, 1 ml (APL, Sweden).

### Treatments and application schedule

The time of suture placement was designated as 0 h. Sutured animals were randomly assigned to one of three topical treatment groups:

1. Rat-specific goat polyclonal pan-*Vegf* antibody (20 µg/ml, Cat. No AF 564, R&D Systems, Minneapolis MN, USA).
2. Dexamethasone (1 mg/ml Opnol, Clean Chemical Sweden AB, Borlänge, Sweden).
3. Normal goat IgG antibody (20 μg/ml, Cat. No. 108-C, R&D Systems).

A naïve group consisted of rats whose corneas were not sutured. Eye drops were administered four times a day for two days. At 48 h, image data were collected, and the portion of the cornea between limbus and the suture was harvested for analysis. A total of 22 rats per treatment group were used, with 6 corneas per group used for RNA extraction for microarray, 10 rats per group for immunohistochemistry, and 6 rats per group for pooled Western blot analysis.

### *In vivo* confocal microscopy (IVCM)

IVCM using Heidelberg Retinal Tomograph 3 with Rostock Corneal Module (Heidelberg Engineering), equipped with a 63x/0.95 NA water-immersion objective (Zeiss, Oberkochen, Germany) was performed in live rat corneas at 48 h, as described elsewhere in detail^39^. Nine representative images of inflammatory cells from the limbal region, from the sutured area and the stromal region between the limbus and suture (no limbus or suture visible) were chosen from each cornea (n = 8–13 corneas per treatment group), for analysis of inflammatory cell infiltration. Cells were quantified manually by a masked observer using a semi-quantitative grading scale developed for this purpose^33^. Briefly, a score was assigned to each image, a median value was found for limbal, stromal and sutured area of each cornea and the sum of these values constituted a total score for each cornea used for statistical analysis. To measure and analyze limbal vessel diameter, three IVCM images focused around the limbal arcade, and showing distinct, perfused blood vessels were selected. From the image sequences for each cornea; the diameter of the vessels was then measured manually using ImageJ (ImageJ software, National Institutes of Health, Bethesda, http://rsb.info.nih.gov/ij/index.html); three independent masked observers performed at least three measurements per image, and the mean of all measurements was used for statistical comparison.

### Aqueous humor collection, cell quantification, and analysis

Aqueous humor (AH) was collected from the anterior chamber using a 30-gauge needle, immediately after sacrificing the animal. A total of approximately 30 µl of raw AH was collected from each eye. The number of cells present in raw AH was determined using the Countess II automated cell counter (Fisher Scientific, Göteborg, Sweden), and cell images were taken using the same instrument.

For CD45 staining, AH cells were fixed with methanol (Sigma-Aldrich, St. Louis, MO, USA, ratio:1:1). Samples were treated with blocking solution (1% BSA/PBS) and stained with primary antibody for CD45 (ab10558, Lot: GR269008-1,1:200, Abcam, Cambridge, United Kingdom). Visualization was achieved with DyLight 488 (1:1000, Thermo Fisher Scientific, Waltham, MA, USA) and mounted with ProLong Diamond Antifade media with DAPI (Invitrogen, Thermo Fisher Scientific, Waltham, MA, USA). Images were taken with an LSM 700 upright confocal microscope (Carl Zeiss AG, Oberkochen, Germany).

### RNA extraction

Following euthanasia, the area of the cornea between the sutures and temporal limbus (corresponding to about 3–4 clock hours of the cornea) was harvested without limbal rim and immersed into RNA later (Qiagen). Total RNA from each sample was isolated with the RNeasy Mini Kit (Qiagen). RNA concentrations were determined using a Nanodrop ND-2000 spectrophotometer (Thermo Scientific). RNA quality was determined using the Agilent Bioanalyzer 2100 (Agilent Technologies Inc., Paolo Alto, CA, USA) and RNA integrity number (RIN) of ≥ 7 was the cut-off for inclusion in microarray analysis.

### Microarray target preparation and hybridization

Of the six RNA samples extracted, four per group (naïve, IgG, anti-*Vegf*, dexamethasone) meeting the RIN inclusion criteria described above were used for microarray target preparation. In total 16 microarray chips were used, one for each cornea. Using 100 ng of total RNA per sample, targets for microarray hybridization were prepared according to the manufacturer’s instructions (GeneChip 3′ IVT Express Kit, Affymetrix.Inc).

### Normalization and analysis of microarray data

Raw cell files were converted into expression measures, background-corrected, and data-normalized using Affymetrix expression console and Affymetrix transcription console (TCA) v. 3.0. The expression data were then transformed to base 2 logarithmic values. Differentially expressed genes (DEGs) were obtained filtering the transformed data for a q-value < 0.05 and fold change (FC) ≤ −1.5 or ≥ 1.5, relative to the naïve corneas. DEGs were then compared among treatment groups, to identify genes unique to treatments. The DEGs were used for biological process overrepresentation analysis with cystoscope BINGO, using a hypergeometric test and the Benjamini & Hochberg False Discovery Rate (FDR) correction^67^, with the significance level set at 0.05. The significantly enriched and overrepresented biological processes were identified, and selected processes were further screened for the genes involved.

The DEGs were used for pathway enrichment analysis using String Software^68^ with an FDR cut-off of 0.05 for significance. From the enriched pathways, those related to inflammation, angiogenesis, and signaling cascades in angiogenesis and the complement system were selected. These pathways were then compared across treatments.

### Real-time reverse transcriptase PCR analysis of selected factors

For real-time reverse transcriptase PCR (qPCR), total RNA was extracted from individual corneal samples (i.e. no pooling) as described above. Complementary DNA was synthesized using SuperScript VILO cDNA Synthesis Kit (Invitrogen). Gene expression analysis was performed using custom TaqMan assays for *Vegfa* and *C1*. For *C3*, SYBR green assay and the following primer sequences were used C3-Forward: GGAGAAAAGCCCAATACCAGC; C3-Reverse: GCAGCCGAAAACCACCATT. Relative CT method was used to determine fold change expression.

### Immunohistochemistry

The area of the cornea between the sutures and temporal limbus was harvested and fixed with 4% paraformaldehyde (Histolab, Gothenburg, Sweden) for 24 h, and embedded in paraffin. Sagittal sections of 5-µm thickness were prepared for immunohistochemical staining with primary antibodies for *Ccl2* (ORB36895; 1:200; Biorbyt, CA, Berkeley, US), *Reg3g* (ORB252969; 1:200 Biorbyt), *Cxcl5* (ORB13450; Biorbyt), *C1s* (BS-15088R; 1:200; Bioss antibodies, MA, Woburn), *Ctgf* (23936-1-AP; 1:200; PTGLab, Rosemont, IL, USA), *C3* (BT-BS8557; 1:1000; Nordic BioSite, Plymouth Meeting, PA, USA); *Vegfa* (GTX21316; 1:250; GeneTex, Simpson, PA, United States) and *CD45* (AB10558; 1:200; Abcam, Cambridge, United Kingdom). Visualization was achieved with DyLight 488 (1:500; Thermo Fisher Scientific, Waltham, MA, USA). Samples were mounted with ProLong Gold antifade (Invitrogen, Thermo Fisher Scientific, Waltham, MA, USA) and imaged using an LSM700 confocal microscope (Carl Zeiss AG, Oberkochen, Germany). CD45 positive cells in corneal fluorescent images were quantified manually by two masked observers and results were averaged. For cornea whole mount immunostaining, fixed corneas were permeabilized by incubation with precooled acetone at −20 °C, washed 3 times with PBS-Tween 20 (PBS-T) 0.1%, blocked overnight with Bovine serum albumin (BSA) (Sigma) 1% at 4 °C, then incubated overnight at 4 °C with rabbit primary antibodies for CD31 (ab28364; 1:100 Abcam), following PBS-T washing and incubation for 1 h at RT with anti-rabbit secondary DyLight 488 (1:500; Thermo Fisher Scientific). Cell nuclei were counterstained by incubation with DAPI (Sigma) 1:1000 for 1 h. Stained corneas were mounted with Dako S3023 fluorescent mounting medium (Agilent Technologies, Santa Clara, United States). Images were acquired using an LSM700 confocal microscope (Carl Zeiss AG, Oberkochen, Germany).

### Western Blot analysis

Corneal samples were harvested as described above, and three samples per group were pooled for Western blot analysis. Samples were homogenized on ice using Qiagen TissueLyser LT (Qiagen, Venlo, Netherlands) in Bio-Rad Ready-Prep™ total protein extraction kit working solution (Bio-Rad, Hercules, California, U.S.A.), supplemented with Halt™ Protease and Phosphatase Inhibitor Cocktail (Bio-Rad). The homogenates were then centrifuged at 17000 G for 10 minutes. Total protein content was determined using Qubit™ 3.0 Fluorometer (Thermo Fisher Scientific, Waltham, Massachusetts, USA) following the Qubit™ quantification protein assay (Thermo Fisher Scientific). A total of 25 µg of protein was loaded to Mini Protean™ precast acrylamide gels (Bio-Rad). Protein transfer was achieved by Trans-Blot® Turbo™ Western Blot transfer system (Biorad), using settings for mixed molecular weight. Membranes were blocked with 5% skimmed milk (Blotting-grade Blocker, Biorad) and subsequently incubated with mouse monoclonal antibody against *Vegfa* (GTX21316; 1:1000; GeneTex); *C3* (BT-BS8557; 1:1000; Nordic BioSite); *C1s* (BS-15088R; 1:1000; Bioss antibodies), at 4 °C overnight, followed by incubation with a horseradish peroxidase–conjugated goat anti-rabbit (AP307P, 2700944) and anti-mouse (AP308P, 2688593) IgG (1:1000; Merck Millipore, Billerica, Massachusetts, USA). Equal whole protein loading was confirmed by *β-actin* (PA1-21167; 1:2000; Thermo Fisher Scientific). Signals were visualized with chemiluminescence Clarity™ Western ECL substrate (Biorad), according to the manufacturer’s protocol using the ImageQuant LAS 500 Imaging System (GE Healthcare Life Sciences, Pittsburgh, PA, US).

### Statistical analysis

For IVCM data, differences in infiltrating cells and limbal vessel dilation were compared across treatments using ANOVA, with the Holm-Sidak multiple comparison methods to isolate pairwise differences. A two-tailed alpha level of < 0.05 was considered significant. SigmaStat 3.5 for Windows (Systat Software Inc., Chicago, USA) used for analysis.

## Electronic supplementary material

Supplementary Information
