# Supplementary material for: Genome-wide expression differences in anti-Vegf and dexamethasone treatment of inflammatory angiogenesis in the rat cornea
Source: Sci Rep. 2017 Aug 15;7:7616. doi: 10.1038/s41598-017-07129-4 (PMC5557983; doi:10.1038/s41598-017-07129-4)
Supplement: Supplementary file 1 — Supplementary Information [file 41598_2017_7129_MOESM1_ESM.doc]

**Supplementary Material**

**Genome-wide expression differences in anti-*Vegf* and dexamethasone treatment of inflammatory angiogenesis in the rat cornea**

Pierfrancesco Mirabelli^#^, Anthony Mukwaya^#^, Anton Lennikov, Maria Xeroudaki, Beatrice Peebo, Mira Schaupper^1^ and Neil Lagali^1^*

Department of Ophthalmology, Institute for Clinical and Experimental Medicine,

Faculty of Health Sciences, Linkoping University, 58183 Linköping, Sweden

^#^Equal contributions

*Corresponding author:

Neil Lagali, PhD

Department of Ophthalmology

Institute for Clinical and Experimental Medicine

Faculty of Health Sciences

Linkoping University,

58183 Linköping, Sweden

Tel +46 101034658

Fax +46 101033065

neil.lagali@liu.se

**Supplementary Figure 1.** Inflammatory cell invasion 48h after corneal injury is not suppressed in any treatment group. (**A**) Immunofluorescence for CD45 (green) and DAPI visualised nuclei (blue) in the rat cornea; (**B**) In vivo images of corneal stromal cellular infiltration by IVCM, and the corresponding quantification in (**E**); n=8, 8, 13 rats in IgG, anti-Vegf and dex groups respectively; no significant difference between groups was observed (P > 0.05); error bars represent SD. (**C**) Aqueous humor inflammatory cells stained by immunofluorescence for CD45 (green) with nuclei stained with DAPI (blue). (**D**) Quantification of CD45+ cell infiltration in the different treatment groups from corneal sections.

**
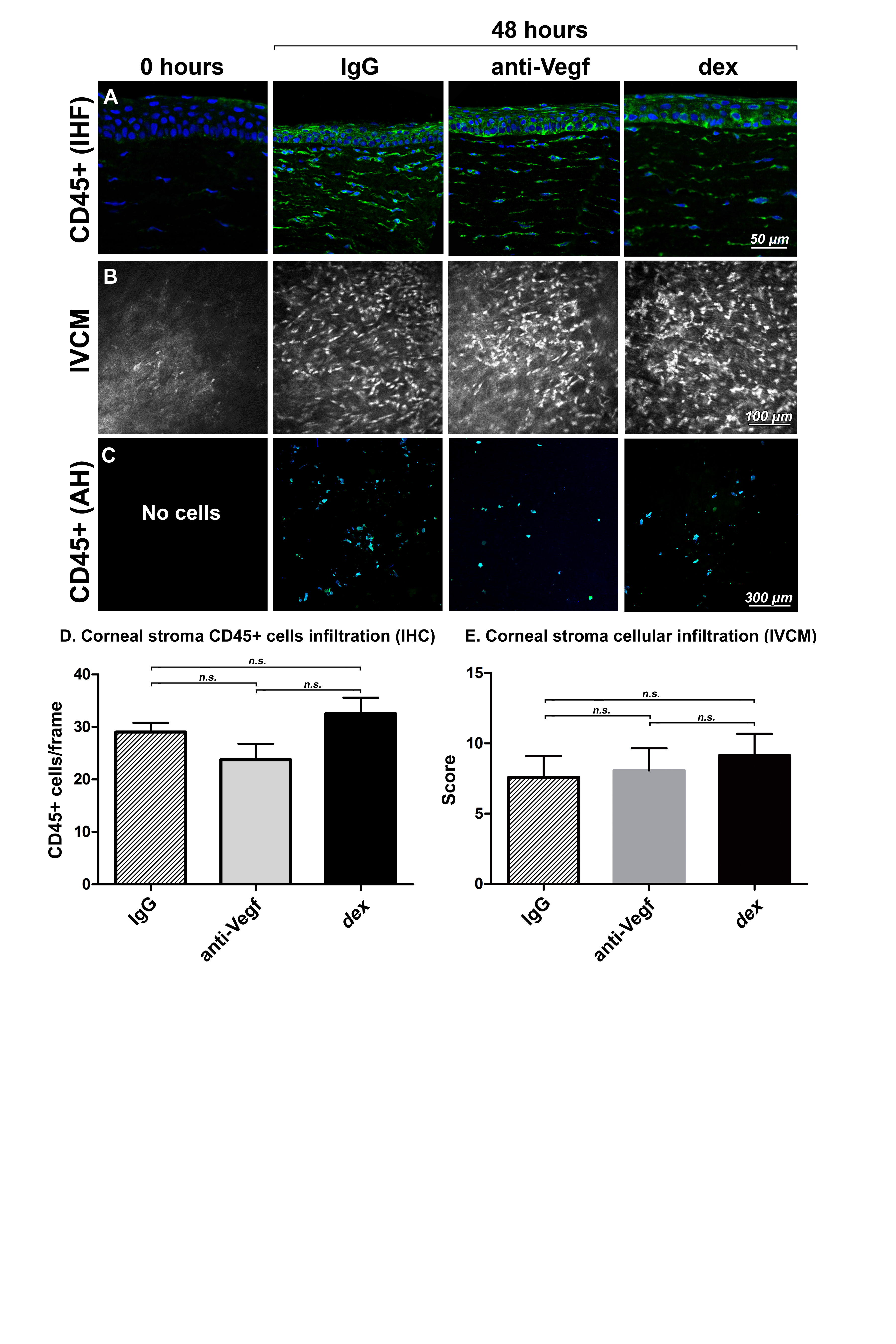
**

**Supplementary Figure 2.** Western blot analysis of selected factors indicating total protein expression in the corneal tissue. (**A**) Expression of *C3* additionally indicating multiple related protein bands; (**B**) Expression of *Vegfa* and *C1s*.

**
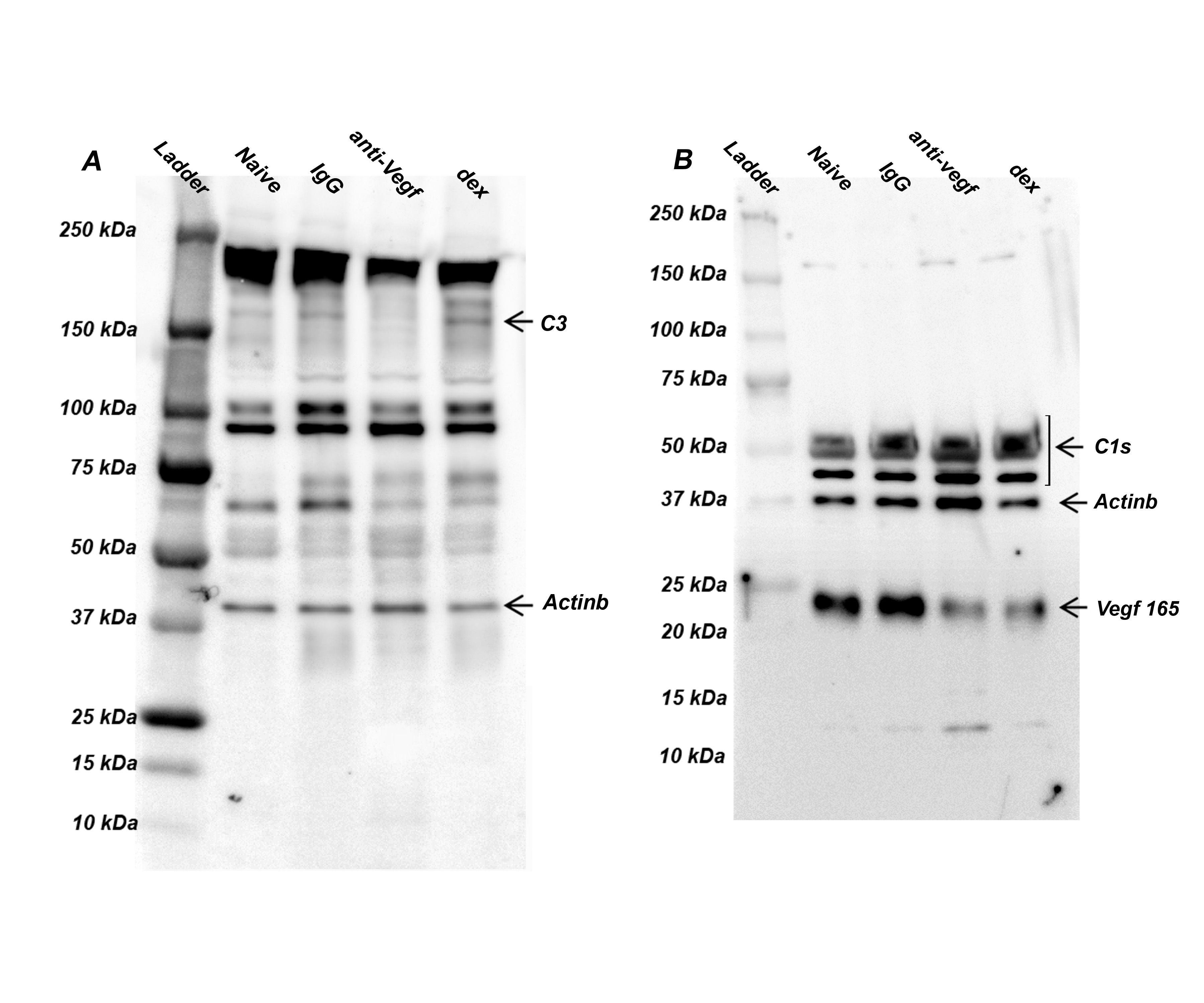
**

**Supplementary Table 1.** Selected pathways, the number of genes involved in them, and the corresponding false discovery rate (FDR).

|  | **IgG** | | **Dexamethasone** | | **Anti-*Vegf*** | |
| --- | --- | --- | --- | --- | --- | --- |
| Pathway description | Count | FDR | Count | FDR | Count | FDR |
| PI3K-Akt signaling pathway | 67 | 2.23E-10 | 50 | 9.69E-07 | 64 | 2.29E-08 |
| Focal adhesion | 45 | 3.28E-08 | 32 | 5.68E-05 | 47 | 2.29E-08 |
| ECM-receptor interaction | 26 | 5.72E-08 | 21 | 1.99E-06 | 28 | 2.29E-08 |
| Chemokine signaling pathway | 36 | 1.70E-06 | 30 | 1.17E-05 | 41 | 4.30E-08 |
| Cytokine-cytokine receptor interaction | 43 | 3.77E-07 | 40 | 1.02E-07 | 44 | 8.18E-07 |
| Complement and coagulation | 21 | 1.31E-06 | 19 | 1.68E-06 | 20 | 1.29E-05 |
| NF-kappa B signaling pathway | 25 | 3.77E-07 | 21 | 2.73E-06 | 22 | 3.39E-05 |
| TNF signaling pathway | 24 | 4.06E-05 | 22 | 2.39E-05 | 23 | 2.98E-04 |
| Leukocyte transendothelial migration | 23 | 4.86E-04 | 21 | 2.56E-04 | 22 | 2.47E-03 |
| Toll-like receptor signaling pathway | 16 | 1.15E-02 | 14 | 1.29E-02 | 17 | 9.33E-03 |
| NOD-like receptor signaling pathway | 13 | 2.17E-03 | 10 | 1.45E-02 | 13 | 3.79E-03 |
| Cell adhesion molecules (CAMs) | 27 | 7.72E-04 | 18 | 4.75E-02 | 25 | 7.33E-03 |

**Supplementary Table 2.** Extended list of genes involved in selected biological processes (a), and in selected pathways (b). ’FC Diff’ represents fold change in dexamethasone minus fold change in anti-*Vegf* group. P-values are for comparisons between dexamethasone and anti-*Vegf* groups.

| **a: From biological process enrichment analysis** | | | | |  | **b: From pathway enrichment analysis** | | | | |
| --- | --- | --- | --- | --- | --- | --- | --- | --- | --- | --- |
| **Gene ID** | **FC: dex** | **FC:**  **anti-*Vegf*** | **FC: Diff** | **P-value** |  | **Gene ID** | **FC: dex** | **FC: anti-*Vegf*** | **FC: Diff** | **P-value** |
| **Differentially upregulated by dexamethasone** | | | | |  | **Differentially upregulated by dexamethasone** | | | | |
| *C3* | 22.12 | 5.56 | 16.56 | 1.04E-02 |  | *C3* | 22.12 | 5.56 | 16.56 | 1.04E-02 |
| *Ctgf* | 1.31 | -2.95 | 4.26 | 2.04E-03 |  | *Fcgr2b* | 14.14 | 9.21 | 4.93 | 3.95E-02 |
| *C1s* | 8.50 | 4.39 | 4.11 | 2.25E-03 |  | *C1s* | 8.50 | 4.39 | 4.11 | 2.25E-03 |
| *Serping1* | 8.34 | 5.00 | 3.34 | 1.41E-02 |  | *Serping1* | 8.34 | 5.00 | 3.34 | 1.41E-02 |
| *Tgfb2* | 1.06 | -1.92 | 2.99 | 3.27E-03 |  | *Tgfb2* | 1.06 | -1.92 | 2.99 | 3.27E-03 |
| *Pou3f3* | 1.13 | -1.61 | 2.74 | 1.12E-03 |  | *Fgfr1* | 1.64 | -1.12 | 2.76 | 2.60E-05 |
| *Tfrc* | 3.64 | 1.80 | 1.84 | 2.76E-03 |  | *Mpzl1* | 1.09 | -1.59 | 2.69 | 1.77E-03 |
| *Enpp2* | -1.17 | -2.72 | 1.55 | 1.61E-02 |  | *Pdgfa* | 1.56 | -1.01 | 2.58 | 1.08E-04 |
| *Cd36* | 3.35 | 1.82 | 1.53 | 7.12E-03 |  | *Mmp9* | 4.02 | 1.92 | 2.09 | 8.22E-03 |
| *Cdh2* | -1.39 | -2.79 | 1.40 | 4.78E-03 |  | *C1r* | 4.88 | 3.16 | 1.71 | 6.38E-03 |
| *Col11a1* | -1.07 | -2.42 | 1.36 | 3.85E-03 |  | *Col6a1* | -1.20 | -2.81 | 1.62 | 1.28E-03 |
| *Steap3* | 2.94 | 1.60 | 1.34 | 3.62E-04 |  | *Cd36* | 3.35 | 1.82 | 1.53 | 7.12E-03 |
| *Ednrb* | -2.43 | -3.71 | 1.28 | 4.70E-02 |  | *Cdh2* | -1.39 | -2.79 | 1.40 | 4.78E-03 |
| *Sema3c* | -1.55 | -2.80 | 1.26 | 6.41E-04 |  | *Col11a1* | -1.07 | -2.42 | 1.36 | 3.85E-03 |
| *Col4a1* | -1.30 | -2.54 | 1.24 | 9.16E-03 |  | *Col4a1* | -1.30 | -2.54 | 1.24 | 9.16E-03 |
| *Nfkbia* | 2.95 | 1.75 | 1.20 | 1.30E-03 |  | *Nfkbia* | 2.95 | 1.75 | 1.20 | 1.30E-03 |
| *Lamb3* | 3.27 | 2.10 | 1.17 | 3.77E-02 |  | *Lamb3* | 3.27 | 2.10 | 1.17 | 3.77E-02 |
| *Col5a1* | -1.65 | -2.81 | 1.16 | 1.50E-02 |  | *Col5a1* | -1.65 | -2.81 | 1.16 | 1.50E-02 |
| *Cadm1* | -1.63 | -2.79 | 1.16 | 1.02E-03 |  | *Cadm1* | -1.63 | -2.79 | 1.16 | 1.02E-03 |
| *Itgbl1* | -1.25 | -2.24 | 0.99 | 3.12E-02 |  | *Col4a5* | -1.35 | -2.49 | 1.14 | 7.01E-03 |
| *Aldh1a1* | -1.68 | -2.65 | 0.96 | 1.22E-02 |  | *Il22ra2* | 2.10 | 1.25 | 0.85 | 2.99E-02 |
| *Sned1* | -1.38 | -2.32 | 0.94 | 5.92E-03 |  | *Cxcr7* | -1.30 | -2.11 | 0.81 | 2.53E-03 |
| *Fstl3* | 2.58 | 1.66 | 0.92 | 2.72E-02 |  | *Thbs2* | 1.79 | 1.02 | 0.77 | 4.24E-03 |
| *Timp3* | -1.32 | -2.22 | 0.90 | 3.23E-02 |  | *Sv2b* | -1.55 | -2.22 | 0.68 | 7.31E-03 |
| *Apod* | 4.12 | 3.31 | 0.81 | 2.98E-02 |  | *Col4a2* | -1.19 | -1.86 | 0.67 | 6.91E-04 |
| *Anpep* | 2.93 | 2.18 | 0.76 | 4.79E-02 |  | *Jam3* | -1.10 | -1.76 | 0.66 | 1.63E-03 |
| *Rarb* | -1.02 | -1.76 | 0.74 | 1.27E-03 |  | *Pvrl3* | 1.90 | 1.27 | 0.63 | 2.38E-02 |
| *Postn* | -1.14 | -1.81 | 0.67 | 3.80E-02 |  | *Rock2* | -1.38 | -1.95 | 0.57 | 6.45E-03 |
| *Pitx2* | -1.17 | -1.79 | 0.62 | 5.71E-03 |  | *Masp1* | 1.77 | 1.20 | 0.56 | 2.66E-02 |
| *Grik5* | -1.05 | -1.66 | 0.61 | 2.02E-02 |  | *Ccl17* | 1.70 | 1.15 | 0.55 | 4.20E-02 |
| *Arhgap24* | -1.56 | -2.15 | 0.59 | 3.36E-02 |  | *Jam2* | -1.60 | -2.15 | 0.55 | 3.06E-02 |
| *Ptprk* | -1.06 | -1.61 | 0.55 | 3.63E-02 |  | *Itgb3* | 1.76 | 1.21 | 0.54 | 3.29E-02 |
| *Ntm* | -1.15 | -1.68 | 0.53 | 1.94E-02 |  | *Gnb1* | -1.21 | -1.76 | 0.54 | 6.83E-04 |
| *Vav2* | -1.20 | -1.73 | 0.53 | 3.50E-03 |  | *Lama5* | 1.78 | 1.25 | 0.53 | 4.46E-03 |
| *Apoe* | -1.12 | -1.63 | 0.51 | 3.27E-03 |  | *Vav2* | -1.20 | -1.73 | 0.53 | 3.50E-03 |
| *Ncdn* | -1.03 | -1.53 | 0.50 | 2.08E-03 |  | *Grk5* | 2.06 | 1.54 | 0.52 | 7.74E-03 |
| *Ass1* | -1.33 | -1.82 | 0.49 | 1.79E-02 |  | *Tnfsf12* | 1.57 | 1.06 | 0.51 | 1.81E-03 |
| *Zfp423* | -1.27 | -1.76 | 0.49 | 4.90E-02 |  | *Flna* | 1.81 | 1.33 | 0.48 | 1.24E-02 |
| *Ccnd1* | -1.61 | -2.05 | 0.45 | 3.05E-02 |  | *Ccnd1* | -1.61 | -2.05 | 0.45 | 3.05E-02 |
| *Clic4* | 2.01 | 1.62 | 0.39 | 2.39E-02 |  | *Lamc1* | 1.71 | 1.28 | 0.43 | 1.91E-02 |
| *Mgp* | 2.73 | 2.34 | 0.39 | 7.21E-03 |  | *Sdc4* | 1.64 | 1.23 | 0.41 | 6.67E-03 |
| *Cdh11* | -1.26 | -1.65 | 0.39 | 3.62E-02 |  | *Rasgrf1* | -1.45 | -1.83 | 0.38 | 3.04E-02 |
| *Rasgrf1* | -1.45 | -1.83 | 0.38 | 3.04E-02 |  | *Il6st* | 1.55 | 1.22 | 0.33 | 1.02E-02 |
| *Arhgef3* | -1.59 | -1.94 | 0.35 | 4.98E-02 |  | *Hsp90b1* | 1.75 | 1.44 | 0.32 | 1.33E-03 |
| *Ldha* | 1.84 | 1.52 | 0.32 | 1.29E-02 |  | *Gnb4* | 1.75 | 1.46 | 0.29 | 4.67E-02 |
| *Inpp5e* | -1.21 | -1.53 | 0.32 | 3.56E-03 |  | *Traf3* | 1.51 | 1.25 | 0.26 | 1.12E-02 |
| *Adnp* | -1.26 | -1.57 | 0.31 | 2.16E-03 |  | *Gnai2* | 1.51 | 1.37 | 0.14 | 3.53E-03 |
| *Bag3* | -1.22 | -1.52 | 0.30 | 2.89E-02 |  | **Differentially downregulated by dexamethasone** | | | | |
| *Maged1* | -1.26 | -1.51 | 0.26 | 2.92E-02 |  | *Ccl2* | 66.17 | 104.94 | -38.76 | 1.37E-02 |
|  |  |  |  |  |  | *Cxcl5* | 6.89 | 14.53 | -7.64 | 3.33E-02 |
| **Differentially downregulated by dexamethasone** | | | | |  | *Cfi* | 3.95 | 8.64 | -4.69 | 1.25E-02 |
| *Ccl2* | 66.17 | 104.94 | -38.76 | 1.37E-02 |  | *Pik3r3* | -1.52 | 1.20 | -2.73 | 1.02E-05 |
| *Reg3g* | 2.65 | 18.18 | -15.53 | 3.35E-03 |  | *Prkx* | -1.02 | 1.56 | -2.58 | 3.38E-03 |
| *Cxcl5* | 6.89 | 14.53 | -7.64 | 3.33E-02 |  | *Cntf* | 1.30 | 2.46 | -1.16 | 8.44E-03 |
| *Cfi* | 3.95 | 8.64 | -4.69 | 1.25E-02 |  | *Cldn1* | 1.38 | 2.51 | -1.13 | 3.98E-03 |
| *Klk6* | -1.04 | 2.10 | -3.14 | 1.49E-02 |  | *C1qa* | 2.55 | 3.62 | -1.07 | 4.16E-02 |
| *Cast* | -1.20 | 1.78 | -2.98 | 2.77E-04 |  | *Nox1* | -3.88 | -2.82 | -1.07 | 3.47E-02 |
| *Adam10* | -1.02 | 1.96 | -2.98 | 3.18E-03 |  | *Ccnd2* | 1.94 | 2.83 | -0.89 | 1.23E-02 |
| *Klk8* | 1.06 | 3.96 | -2.90 | 5.25E-03 |  | *Itga6* | 1.88 | 2.74 | -0.86 | 1.65E-02 |
| *Ehf* | -1.11 | 1.68 | -2.79 | 4.66E-04 |  | *C1qc* | 2.02 | 2.79 | -0.78 | 2.92E-02 |
| *Trim54* | -1.08 | 1.71 | -2.79 | 2.28E-03 |  | *Bcl2l1* | 1.09 | 1.78 | -0.69 | 4.39E-02 |
| *Rest* | -1.08 | 1.67 | -2.75 | 1.50E-03 |  | *Msn* | 1.79 | 2.46 | -0.67 | 4.99E-02 |
| *Hdac1* | -1.10 | 1.62 | -2.72 | 2.06E-03 |  | *Ifnar1* | 1.67 | 2.22 | -0.56 | 2.67E-02 |
| *LOC501546* | -1.02 | 1.67 | -2.69 | 1.53E-03 |  | *Marcks* | 1.01 | 1.53 | -0.52 | 2.35E-02 |
| *Psen1* | -1.08 | 1.58 | -2.66 | 2.69E-03 |  | *Gngt2* | 1.70 | 2.19 | -0.49 | 3.28E-02 |
| *Srf* | -1.01 | 1.58 | -2.59 | 1.67E-03 |  | *Ocln* | -1.63 | -1.15 | -0.48 | 4.93E-03 |
| *Sox7* | 5.35 | 7.66 | -2.32 | 1.01E-02 |  | *Traf6* | 1.05 | 1.52 | -0.47 | 1.25E-02 |
| *Gap43* | -3.60 | -2.17 | -1.43 | 1.97E-03 |  | *Casp7* | 1.20 | 1.65 | -0.45 | 4.60E-02 |
| *Sprr1b* | 1.14 | 2.54 | -1.40 | 6.25E-03 |  | *Nfkb1* | 1.70 | 2.10 | -0.40 | 1.63E-02 |
| *Casp12* | 1.57 | 2.87 | -1.30 | 4.08E-03 |  | *Pten* | 1.24 | 1.60 | -0.36 | 6.17E-03 |
| *Zeb2* | 2.12 | 3.40 | -1.28 | 5.34E-03 |  | *Ppp2r5c* | 1.26 | 1.60 | -0.34 | 1.81E-03 |
| *Cntf* | 1.30 | 2.46 | -1.16 | 8.44E-03 |  | *Stat1* | 1.26 | 1.57 | -0.31 | 5.05E-03 |
| *Cldn1* | 1.38 | 2.51 | -1.13 | 3.98E-03 |  | *Itgav* | 1.49 | 1.78 | -0.29 | 4.67E-02 |
| *C1qa* | 2.55 | 3.62 | -1.07 | 4.16E-02 |  | *Mllt4* | -1.55 | -1.32 | -0.23 | 3.50E-02 |
| *Elk3* | 1.30 | 2.28 | -0.97 | 2.25E-04 |  |  |  |  |  |  |
| *Ccnd2* | 1.94 | 2.83 | -0.89 | 1.23E-02 |  |  |  |  |  |  |
| *Nedd4* | 1.06 | 1.94 | -0.88 | 1.61E-03 |  |  |  |  |  |  |
| *Itga6* | 1.88 | 2.74 | -0.86 | 1.65E-02 |  |  |  |  |  |  |
| *Acp5* | 1.60 | 2.39 | -0.79 | 2.77E-03 |  |  |  |  |  |  |
| *C1qc* | 2.02 | 2.79 | -0.78 | 2.92E-02 |  |  |  |  |  |  |
| *Pdlim5* | 1.02 | 1.80 | -0.77 | 4.48E-04 |  |  |  |  |  |  |
| *Bcl2l1* | 1.09 | 1.78 | -0.69 | 4.39E-02 |  |  |  |  |  |  |
| *Msn* | 1.79 | 2.46 | -0.67 | 4.99E-02 |  |  |  |  |  |  |
| *Rdh10* | 1.65 | 2.30 | -0.65 | 2.88E-03 |  |  |  |  |  |  |
| *Tyms* | 1.85 | 2.46 | -0.61 | 2.97E-02 |  |  |  |  |  |  |
| *Rassf6* | 1.44 | 2.03 | -0.59 | 1.02E-02 |  |  |  |  |  |  |
| *Sp110* | 1.08 | 1.66 | -0.57 | 1.12E-02 |  |  |  |  |  |  |
| *Il7* | 1.19 | 1.72 | -0.54 | 9.74E-03 |  |  |  |  |  |  |
| *Tbl1x* | 1.11 | 1.64 | -0.53 | 5.02E-03 |  |  |  |  |  |  |
| *Rnf114* | 1.03 | 1.54 | -0.51 | 2.75E-02 |  |  |  |  |  |  |
| *Dnajb6* | 1.20 | 1.70 | -0.50 | 1.10E-03 |  |  |  |  |  |  |
| *Traf6* | 1.05 | 1.52 | -0.47 | 1.25E-02 |  |  |  |  |  |  |
| *Sp1* | 1.11 | 1.58 | -0.47 | 2.65E-02 |  |  |  |  |  |  |
| *Usp7* | 1.13 | 1.57 | -0.44 | 2.11E-02 |  |  |  |  |  |  |
| *Bmp3* | 1.49 | 1.93 | -0.44 | 1.92E-02 |  |  |  |  |  |  |
| *Pi4k2a* | 1.09 | 1.50 | -0.42 | 8.37E-03 |  |  |  |  |  |  |
| *Ptafr* | 1.14 | 1.55 | -0.41 | 4.27E-02 |  |  |  |  |  |  |
| *Foxo1* | 1.12 | 1.53 | -0.41 | 5.65E-03 |  |  |  |  |  |  |
| *Nfkb1* | 1.70 | 2.10 | -0.40 | 1.63E-02 |  |  |  |  |  |  |
| *Tcp11* | 1.14 | 1.51 | -0.37 | 6.63E-03 |  |  |  |  |  |  |
| *Pten* | 1.24 | 1.60 | -0.36 | 6.17E-03 |  |  |  |  |  |  |
| *Dnajc5* | 1.15 | 1.51 | -0.36 | 7.07E-03 |  |  |  |  |  |  |
| *Dicer1* | 1.20 | 1.54 | -0.34 | 8.46E-03 |  |  |  |  |  |  |
| *Galnt2* | 1.29 | 1.63 | -0.34 | 3.96E-02 |  |  |  |  |  |  |
| *Stat1* | 1.26 | 1.57 | -0.31 | 5.05E-03 |  |  |  |  |  |  |
| *Itgav* | 1.49 | 1.78 | -0.29 | 4.67E-02 |  |  |  |  |  |  |
| *Gfra3* | 1.32 | 1.60 | -0.28 | 3.82E-02 |  |  |  |  |  |  |

**Supplementary Table 3:** Top twenty differentially up- and downregulated genes arranged by descending fold change difference (dexamethasone minus IgG). Genes were selected from those differentially dysregulated by dexamethasone relative to IgG, in the whole genome analysis.

| **Top 20 genes upregulated by dexamethasone** | | | | |
| --- | --- | --- | --- | --- |
| **Gene ID** | **FC: dex** | **FC:IgG** | **FC: Diff** | **P-Value** |
| *C3* | 22.12 | 6.79 | 15.33 | 1.59E-02 |
| *Cp* | 11.96 | 3.64 | 8.32 | 1.14E-02 |
| *C2* | 8.56 | 2.62 | 5.95 | 1.74E-02 |
| *Mt2A* | 12.81 | 7.22 | 5.59 | 8.81E-04 |
| *Mt1a* | 10.92 | 5.69 | 5.23 | 3.09E-03 |
| *Wfdc18* | 8.48 | 3.92 | 4.56 | 6.96E-03 |
| *Tsc22d3* | 2.24 | -1.68 | 3.92 | 6.58E-03 |
| *Andpro* | 1.10 | -2.19 | 3.29 | 1.68E-04 |
| *Plce1* | 1.36 | -1.79 | 3.16 | 3.02E-03 |
| *Vps37b* | 1.19 | -1.90 | 3.10 | 1.58E-03 |
| *Klf15* | 1.00 | -2.00 | 3.00 | 4.03E-02 |
| *Zfp367* | 1.19 | -1.75 | 2.94 | 6.00E-04 |
| *C1s* | 8.50 | 5.57 | 2.94 | 1.52E-02 |
| *Wdr67* | 1.08 | -1.85 | 2.93 | 1.65E-02 |
| *Nusap1* | 1.26 | -1.52 | 2.78 | 7.85E-03 |
| *Flrt3* | 1.22 | -1.50 | 2.73 | 8.33E-04 |
| *Col6a2* | 1.03 | -1.65 | 2.69 | 2.38E-04 |
| *Ikbip* | 1.13 | -1.54 | 2.67 | 6.20E-03 |
| *Pou3f3* | 1.13 | -1.54 | 2.67 | 2.00E-03 |
| *Atp1b1* | 1.03 | -1.63 | 2.66 | 2.92E-03 |
| **Top 20 genes downregulated by dexamethasone** | | | | |
| *Serpinb2* | 35.22 | 59.68 | -24.47 | 4.14E-02 |
| *Lrat* | 3.54 | 23.86 | -20.32 | 1.66E-06 |
| *Stfa3* | 5.51 | 25.05 | -19.53 | 2.28E-02 |
| *Reg3g* | 2.65 | 17.30 | -14.65 | 3.15E-02 |
| *Cxcl5* | 6.89 | 19.38 | -12.49 | 7.37E-03 |
| *Stfa2l3* | 9.78 | 19.28 | -9.51 | 1.95E-02 |
| *RT1-A2* | -1.14 | 6.94 | -8.07 | 2.55E-03 |
| *Cfi* | 3.95 | 11.93 | -7.98 | 1.29E-02 |
| *LOC257642* | 1.01 | 6.22 | -5.21 | 1.14E-03 |
| *Dmkn* | 3.10 | 7.87 | -4.77 | 7.09E-04 |
| *Cnfn* | 3.40 | 7.65 | -4.26 | 3.73E-02 |
| *Socs3* | 2.80 | 6.25 | -3.45 | 5.03E-03 |
| *Zbtb41* | -1.17 | 2.20 | -3.38 | 1.40E-04 |
| *LOC100910973* | -1.32 | 2.06 | -3.38 | 3.00E-03 |
| *Rest* | -1.08 | 2.29 | -3.37 | 2.12E-05 |
| *RT1-Da* | 5.68 | 9.01 | -3.33 | 3.77E-02 |
| *Ints6* | -1.24 | 2.09 | -3.33 | 6.38E-05 |
| *Hectd1* | -1.18 | 2.07 | -3.25 | 8.74E-03 |
| *Rab1a* | -1.42 | 1.84 | -3.25 | 3.29E-04 |
| *Gja1* | -1.38 | 1.86 | -3.24 | 3.19E-04 |
